# Human umbilical cord mesenchymal stem cells alleviate brain injury and promote neuroregeneration via BDNF/TrkB/CREB/PI3K-Akt pathway in preterm rats with intraventricular hemorrhage

**DOI:** 10.1093/stcltm/szag086

**Published:** 2026-10-16

**Authors:** Mingyou Yu, Ziyi Zhang, Xuxian Wu, Baoying Zhang, Yang Hu, Changge Wei, Miao Ran, Manni Cao, Jinhui Zhang, Xueli Ding, Yan Wang, Can Liu, Maoqiong Chen, Jianwei Xu

**Affiliations:** Center for Tissue Engineering and Stem Cell Research, Guizhou Medical University, Guiyang 550000, Guizhou, China; Department of Neonatology, Affiliated Hospital of Guizhou Medical University, Guiyang 550000, Guizhou, China; Department of Neonatology, Tongren People’s Hospital, Tongren 554300, Guizhou, China; Center for Tissue Engineering and Stem Cell Research, Guizhou Medical University, Guiyang 550000, Guizhou, China; Center for Tissue Engineering and Stem Cell Research, Guizhou Medical University, Guiyang 550000, Guizhou, China; Center for Tissue Engineering and Stem Cell Research, Guizhou Medical University, Guiyang 550000, Guizhou, China; Center for Tissue Engineering and Stem Cell Research, Guizhou Medical University, Guiyang 550000, Guizhou, China; Department of Neonatology, Affiliated Hospital of Guizhou Medical University, Guiyang 550000, Guizhou, China; Center for Tissue Engineering and Stem Cell Research, Guizhou Medical University, Guiyang 550000, Guizhou, China; Center for Tissue Engineering and Stem Cell Research, Guizhou Medical University, Guiyang 550000, Guizhou, China; Center for Tissue Engineering and Stem Cell Research, Guizhou Medical University, Guiyang 550000, Guizhou, China; Center for Tissue Engineering and Stem Cell Research, Guizhou Medical University, Guiyang 550000, Guizhou, China; Center for Tissue Engineering and Stem Cell Research, Guizhou Medical University, Guiyang 550000, Guizhou, China; Center for Tissue Engineering and Stem Cell Research, Guizhou Medical University, Guiyang 550000, Guizhou, China; Guizhou Guizhong Biotechnology Co., Ltd., Guiyang 550000, Guizhou, China; Department of Neonatology, Affiliated Hospital of Guizhou Medical University, Guiyang 550000, Guizhou, China; Center for Tissue Engineering and Stem Cell Research, Guizhou Medical University, Guiyang 550000, Guizhou, China; Department of Neonatology, Affiliated Hospital of Guizhou Medical University, Guiyang 550000, Guizhou, China; Department of Pharmacology, School of Basic Medical Sciences, Guizhou Medical University, Guiyang 550000, Guizhou, China

**Keywords:** human umbilical cord-derived mesenchymal stem cells, intraventricular hemorrhage, neuroprotection, signaling pathway, preterm rats

## Abstract

**Objective:**

To explore the neuroprotective effect and molecular mechanism of human umbilical cord mesenchymal stem cells (hUC-MSCs) in preterm rats with intraventricular hemorrhage (IVH), and provide experimental basis for clinical treatment of neonatal IVH.

**Methods:**

Tissue block adherence method was used to isolate and culture hUC-MSCs, and P4 Sprague-Dawley premature rats were divided into sham, IVH model, and hUC-MSC treatment groups. Collagenase VIIS was injected to establish IVH model, and hUC-MSCs were injected into lateral ventricles. Cell colonization, body weight, neurobehavior, brain histology, apoptosis, and related molecular indicators were detected via fluorescence tracking, behavioral tests, staining, RT-PCR, Western blot (WB), and ELISA. Bioinformatics combined with ANA12 blocking assay verified core pathways.

**Results:**

hUC-MSCs survived in rat brain for at least 7 days. Compared with the model group, the treatment group gained weight faster, neurobehavioral function was markedly improved, neuronal apoptosis decreased sharply, and brain tissue damage was alleviated. hUC-MSCs up-regulated anti-apoptotic, anti-inflammatory, antioxidant and neurogenesis-related molecules and activated BDNF/TrkB/CREB/PI3K-Akt pathway; TrkB blockade reversed all protective effects.

**Conclusion:**

hUC-MSCs exert multi-target neuroprotection by activating BDNF/TrkB/CREB/PI3K-Akt pathway, which offers a novel strategy for IVH treatment in preterm infants.

## Graphical abstract

**Graphical Abstract:**
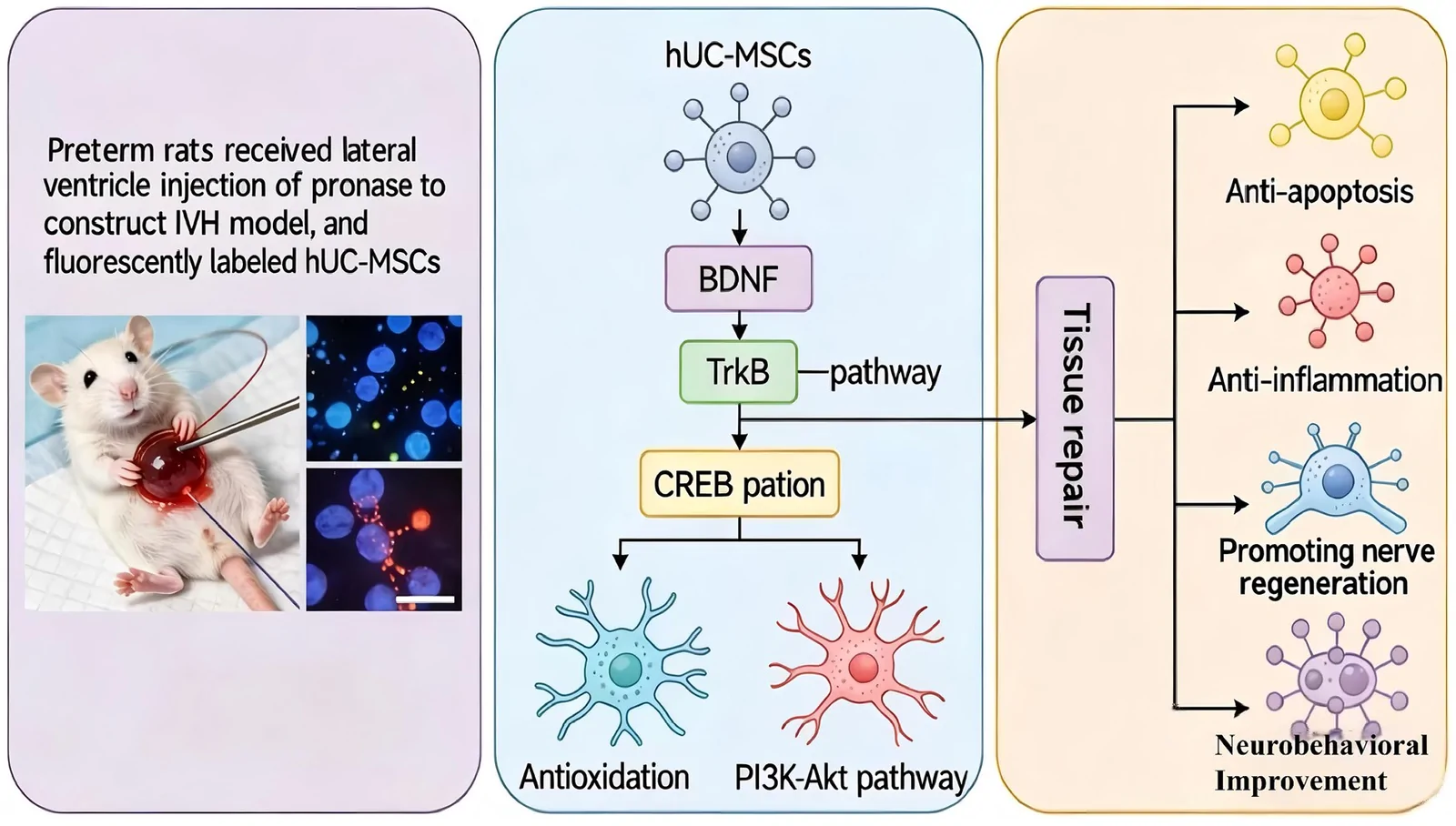

Significance statement Preterm intraventricular hemorrhage (IVH) results in irreversible brain damage with limited therapies. Single medicines cannot resolve IVH-induced inflammation, oxidative stress, and neuronal apoptosis. Human umbilical cord mesenchymal stem cells (hUC-MSCs) survive in injured brain for at least 7 days, alleviating multiple brain injuries via the BDNF/TrkB/CREB/PI3K-Akt pathway. Secreted BDNF activates CREB and PI3K-Akt to suppress apoptosis, inflammation, and glial scarring while promoting neurogenesis. This work clarifies hUC-MSCs’ core neuroprotective mechanism and provides preclinical evidence for treating IVH-related neurological sequelae.

## Introduction

Intraventricular hemorrhage (IVH) is the main type of intracerebral hemorrhage in neonates, defined as the pathological process in which bleeding from the germinal matrix (GM) breaks through the ventricular ependyma into the lateral ventricles. It is most common in premature infants with a gestational age <32 weeks and birth weight <1500 g, with an incidence of 15%-20%; the incidence is as high as 50% in extremely premature infants with a gestational age <28 weeks and birth weight <750 g, making it one of the most common and devastating neurological complications in premature infants.^1^ The risk of IVH is negatively correlated with the gestational age and birth weight of premature infants. Its pathological mechanism is complex: bleeding directly compresses the brain tissue, inducing ischemia-hypoxia, elevated intracranial pressure, and blood–brain barrier impairment. After blood components extravasate, erythrocyte lysis releases thrombin and hemoglobin, which activate microglia to induce inflammatory cascades and generate large amounts of reactive oxygen species (ROS) through the Fenton reaction, leading to oxidative stress injury in neurons and oligodendrocyte progenitor cells.^2^^,^^3^ Ultimately, it can cause sequelae such as white matter injury, hydrocephalus, and brain atrophy, and survivors often suffer from cerebral palsy, cognitive impairment, epilepsy, and other conditions that severely affect quality of life.^4^^,^^5^

Due to the multifactorial complexity of secondary brain injury after IVH, single-drug therapy has limited efficacy, and drug use in premature infants is restricted. Cell transplantation has thus become a potential therapeutic strategy. Mesenchymal stem cells (MSCs) exhibit pleiotropic protective effects, good safety, and easy cultivation. Among them, human umbilical cord mesenchymal stem cells (hUC-MSCs) are isolated from umbilical cord tissue of neonates, with wide availability, no ethical controversy, low immunogenicity, and the ability to avoid immune rejection in autologous transplantation.^6^ Studies have confirmed that MSCs exert anti-inflammatory, anti-oxidative, anti-apoptotic, and neuroregenerative effects through paracrine bioactive factors.^7^^,^^8^ Previous studies by our team have also found that hUC-MSCs can improve neurological function in neonatal rats with hypoxic-ischemic encephalopathy. However, systematic research and mechanistic investigation on IVH in premature rat models remain inadequate, especially regarding the colonization pattern of hUC-MSCs in the brain, direct evidence of apoptosis inhibition, and the synergistic regulatory mechanisms of multiple signaling pathways. Therefore, this study aimed to clarify the protective effects of hUC-MSCs on neurological function and brain tissue morphology in IVH premature rats, and to identify key targets and pathways involved in hUC-MSCs and IVH, providing theoretical basis for clinical translation through the establishment of an IVH model in premature rats, cell fluorescence tracking, immunofluorescence detection, and bioinformatics analysis.

## Methods

### Experimental animals and ethical statement

Specific pathogen-free Sprague-Dawley (SD) rats were provided by the Experimental Animal Center of Guizhou Medical University (license number: SCXK (Xiang) 2019-0014). Four-day-old postnatal (P4) rat pups, weighing 10-12 g (both sexes, no restriction), were randomly grouped and housed in an environment with constant temperature (22 ± 2 °C), constant humidity (50% ± 10%), and a 12 h light/dark cycle, with free access to food and water; all animal experimental protocols were reviewed and approved by the Experimental Animal Ethics Committee of Guizhou Medical University (approval number: 2403736) and complied with the ARRIVE Guidelines.

### Isolation, culture, and identification of hUC-MSCs

Primary hUC-MSCs were isolated using the tissue block adherence method: fresh umbilical cord tissue was aseptically separated, cut into small blocks, and inoculated into culture flasks containing complete medium. The cells were cultured in a 37 °C, 5% CO₂ incubator, and cell migration and growth were observed. When cell confluence reached 80%-90%, the cells were passaged and purified to the fourth passage (P4) for experiments. Flow cytometry was used to detect the expression of surface markers (positive markers CD73, CD90, CD105, and negative markers) in P4 cells; For fluorescent labeling, P4 hUC-MSCs were digested, washed, and stained with CellTracker Green CMFDA, washed repeatedly to remove free dye, and resuspended to 5 × 10^5^ cells/3 μL. Cell viability after labeling was confirmed over 92% by trypan blue staining. hUC-MSCs were fluorescently labeled for subsequent *in vivo* colonization tracking. Three independent human umbilical cord tissue donors were enrolled for hUC-MSC isolation. All 3 donor-derived hUC-MSC batches fulfilled standard MSC phenotypic criteria: spindle-shaped fibroblast-like morphology; flow‑cytometry positivity for CD73, CD90, CD105 (>95%) and negativity for hematopoietic lineage markers (<2%); trypan-blue viability >92% at passage 4. Cells from Donor-2 were selected for all formal *in vivo* animal experiments reported in the article. Donor selection criteria included: (1) high cell viability (>92%) at P4, (2) stable proliferative capacity reaching 80%-90% confluence within consistent culture duration; (3) meeting the surface‑marker threshold for MSCs. Preliminary small‑scale *in vivo* pilot tests were performed for all 3 donor batches; comparable trends for body-weight recovery, neuronal apoptosis suppression and pathway-molecule activation were observed across donor groups, confirming consistent therapeutic performance and excluding major donor‑dependent variability. Formal quantitative main-study data presented in figures are generated using cells from Donor-2.

### Establishment of IVH model and grouping of premature rats

Premature rats were randomly divided into 3 groups (*n* = 10/group, *n* = 3 for molecular biological detection): sham operation group (Sham group), IVH model group (Vehicle group), and hUC-MSC administration group (hUC-MSCs-treated group). The stereotactic injection coordinates (mediolateral [ML]: +1.5 mm, anteroposterior [AP]: +1.6 mm, DV (dorsoventral): 2.3 mm) for germinal matrix hemorrhage (GMH-IVH model) were determined through 3 standardized steps: (1) Coordinate reference: We adopted the neonatal SD rat stereotaxic brain atlas specific for postnatal day 4 (P4) pups as the baseline benchmark, with bregma set as the zero reference point for all anteroposterior (AP) and mediolateral (ML) measurements, and DV depth measured vertically from the skull surface down to the target GM tissue. (2) Pre-experiment calibration: A series of gradient coordinate pretests were conducted on spare P4 rat pups before formal modeling; different AP/ML/DV combinations were trialed, followed by post-injection brain gross dissection and HE staining to compare hemorrhage range, and the coordinate combination producing stable, localized GM bleeding (0.6U collagenase VIIS injection volume) was finalized. (3) Intraoperative positional verification: During each operation, rat pup heads were fixed on a neonatal stereotaxic frame with the cranial sagittal plane kept horizontal; bregma landmark was marked under transillumination of cranial sutures to calibrate the coordinate origin before needle penetration. The GMH-IVH model was established by local injection of collagenase VIIS on P4 (4 days after birth) at the coordinates ML: +1.5 mm, AP: +1.6 mm, DV: 2.3 mm with an injection dose of 0.6U; the Sham group was injected with an equal volume of normal saline. At 48 h post-modeling, the success of the model was verified by gross observation of brain tissues, HE staining, and neurobehavioral assessment. On P6 (2 days post-modeling), the hUC-MSCs-treated group received a lateral ventricular injection of 5 × 10^5^ fluorescently labeled hUC-MSCs (volume: 3 μL) at the coordinates ML: +0.8 mm, AP: +1.0 mm, DV: 2.3 mm; This cell dosage and suspension volume were consistent for all *in vivo* transplantation-related experiments in the present study. A 26 G beveled micro-injection needle matched with 5 μL Hamilton microsyringe was adopted. The suspension was injected at a low rate of 1 μL/min within 3 min, with the needle retained for 2 min after injection to reduce shear stress and cell backflow. The Sham group and Vehicle group were injected with an equal volume of normal saline. An additional ANA12 blocking group was set up, in which the TrkB-specific antagonist ANA12 was injected into the lateral ventricles simultaneously with modeling and hUC-MSC administration. ANA12 was dissolved in sterile DMSO-PBS mixed vehicle, prepared to a final concentration of 100 ng/μL; a total volume of 3 μL ANA12 solution (300 ng total ANA12) was injected via the same 26 G micro-injection needle and identical slow infusion protocol as hUC-MSCs. This dosage was selected based on 2 grounds: (1) published ICV ANA12 regimens in neonatal rat GM hemorrhage models for full TrkB pathway inhibition; (2) preliminary gradient concentration tests (50, 150, 300, 600 ng). A dose of 300 ng/3 μL achieved sufficient TrkB suppression, while higher doses induced nonspecific neuronal injury.

### Detection indicators and methods

#### In vivo fluorescence tracking of hUC-MSCs

Brain tissues of rats in the hUC-MSCs-treated group were collected at 1, 3, and 7 days after hUC-MSC injection, and the colonization, survival, and distribution of hUC-MSCs in the brain were observed by fluorescence microscopy.

#### General condition monitoring

Body weights of rats in each group were recorded on P4, P10, and P14.

#### Neurobehavioral evaluation

Neurological deficit was assessed using the Longa score (P7); vestibular function was assessed via the righting reflex test (P7); motor coordination was evaluated using the geotaxis test (P9, P13) and rotarod test (P27); muscle strength was assessed using the forelimb grip test (P9, P13); and learning/memory function was evaluated using the Morris water maze test (P34).

#### Histological detection

Rats were sacrificed on postnatal day 32 (P32), and brain tissues were harvested for hematoxylin and eosin (HE) staining (to observe the cerebral cortex and canonical hippocampal structure) and Nissl staining (to evaluate neuronal number in the cerebral cortex and canonical hippocampus, as well as ventricular dilatation). Rats were sacrificed on postnatal day 7 (P7), and brain tissues were collected for TUNEL staining (to detect cell apoptosis in brain tissue, including DAPI nuclear staining, TUNEL apoptotic signals, and merged images) and immunofluorescence staining (to detect the localization and expression of p-CREB protein, including DAPI nuclear staining, p-CREB fluorescent signals, and merged images). Quantitative analysis was performed via microscopic observation and image analysis.

#### Bioinformatics analysis

Common targets between hUC-MSCs and IVH were screened, a protein–protein interaction (PPI) network was constructed, and GO functional enrichment analysis (biological processes [BP], cellular components [CC], molecular functions [MF]) and KEGG signaling pathway analysis were performed.

#### Molecular biological detection

Brain tissues surrounding the lateral ventricles were harvested on P7. RT-PCR was used to detect the transcriptional levels of *BDNF*, *CREB*, and *TrkB* genes; Western blot (WB) was used to detect the protein expression of BDNF, Bax, cleaved caspase-3, caspase-3, Bcl-2, glial fibrillary acidic protein (GFAP), Nestin, Tuj1, HO-1, p-CREB, CREB, p-AKT, AKT, p-TrkB, TrkB, and Arg1; colorimetry was used to detect MDA content and the activities of SOD, GSH-PX, and CAT; ELISA was used to detect the contents of IL-6, TNF-α, IL-10, and TGF-β.

#### Signaling pathway blockade assay

After TrkB blockade with ANA12, the expression changes of apoptosis-related proteins (Bax, Bcl-2), neuroregeneration markers (Nestin, Tuj1), signaling pathway molecules (BDNF, p-CREB, p-TrkB, p-AKT), and GFAP were detected to verify the core role of the BDNF/TrkB/CREB/PI3K-Akt pathway.

### Statistical analysis

SPSS 22.0 software was used for statistical analysis. Measurement data were expressed as mean ± SD (x ± s). One-way analysis of variance was performed for comparisons among multiple groups, and the LSD-t test was used for pairwise comparisons between groups. A *P*-value <.05 was considered statistically significant, *P *< .01 was considered very significant, and *P *< .001 was considered extremely significant.

## Results

### Isolation, culture, identification, and fluorescent labeling of hUC-MSCs

On day 14 of culture using the tissue block adherence method, hUC-MSCs migrated out of the tissue blocks and reached over 80% confluence, showing spindle-shaped adherent growth; P4 hUC-MSCs had a uniform morphology, presenting a typical fibroblast-like appearance (Figure 1A and B). Flow cytometry showed that the cells highly expressed the positive markers CD73 (98.8%), CD105 (99.4%), and CD90 (99.9%), while the expression of negative surface markers was only 0.12% (an extremely low level, < 0.1%), consistent with the phenotypic characteristics of MSCs (Figure 1C). The viability of fluorescently labeled hUC-MSCs was not significantly altered, which could be used for *in vivo* tracking experiments.

**Figure 1.**
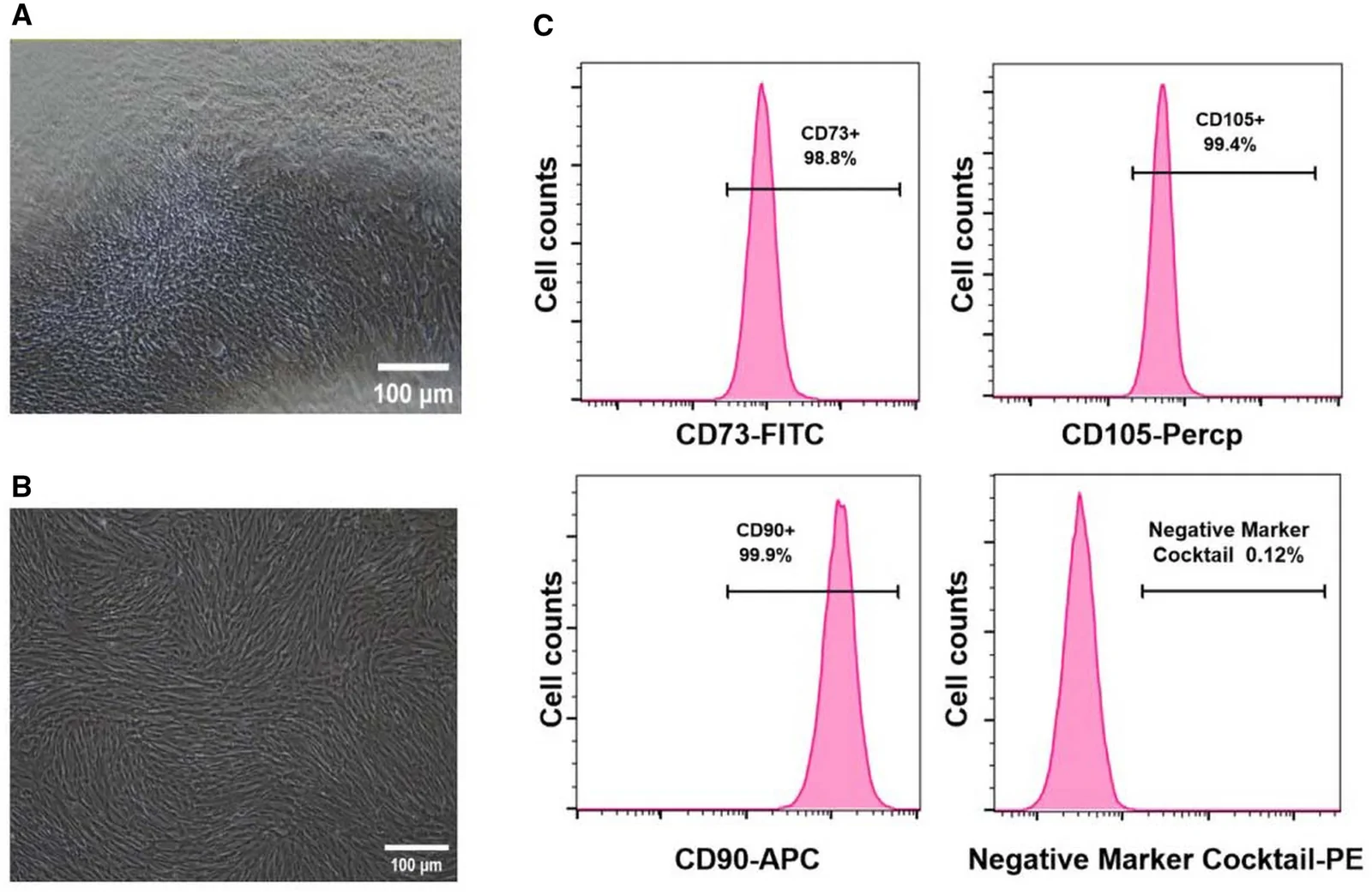
Isolation, purification, and identification of hUC-MSCs. (A) Isolation of primary hUC-MSCs (tissue block adherence method, microscopic image of hUC-MSCs migrating out of tissue blocks on day 14, scale bar = 100μm). (B) Microscopic morphology of purified P4 hUC-MSCs (scale bar = 100 μm). (C) Flow cytometry identification of hUC-MSCs (high expression of CD73, CD105, CD90, low expression of negative markers; labeled with CD73-FITC, CD105-Percp, CD90-APC, and negative markers labeled with Cocktail-PE).

### Establishment, verification of IVH model, and confirmation of treatment method

At 48 h post-modeling, obvious intracerebral hemorrhagic foci were observed in the gross brain specimens and coronal brain sections of rats in the Vehicle group, and HE staining further confirmed the hemorrhage area (Figure 2D); the hemorrhage area in the 0.6U collagenase group (approximately 0.035 mm²) was significantly larger than that in the 0.3U group (approximately 0.015 mm²) (*P *< .01, Figure 2B and C). The injection coordinates for IVH model establishment were ML: +1.5 mm, AP: +1.6 mm, DV: 2.3 mm (Figure 2A), and the coordinates for lateral ventricular injection of hUC-MSCs were ML: +0.8 mm, AP: +1.0 mm, DV: 2.3 mm, with the injection site was verified via Evans blue staining (Figure 2E and F). Neurobehavioral evaluation showed that the Longa neurological deficit score (approximately 1.5 points) of the Vehicle group on day 3 post-modeling was significantly higher than that of the Sham group (0 points) (*P *< .001); the righting reflex time (approximately 9 s) was significantly longer than that of the Sham group (approximately 2 s) (*P *< .01); the geotaxis climbing time (approximately 35 s) on day 5 post-modeling was significantly longer than that of the Sham group (approximately 10 s) (*P *< 0.001); and the forelimb grip time (approximately 2 s) was significantly shorter than that of the Sham group (approximately 8 s) (*P *< .01) (Figure 2G-J), indicating successful establishment of the IVH model, with 0.6U as the optimal modeling dose.

**Figure 2.**
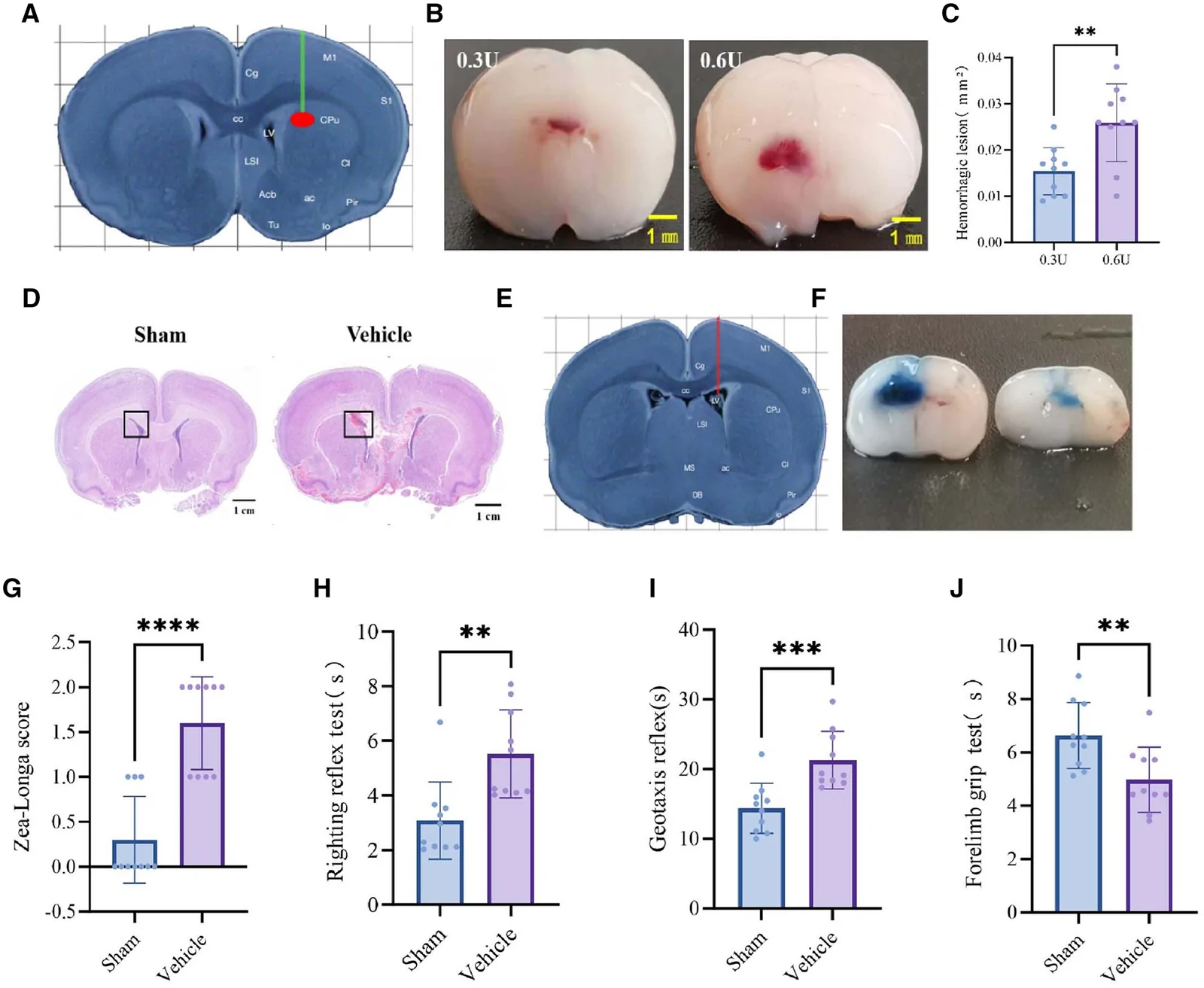
Establishment, verification of IVH model in premature rats, and evaluation of treatment methods and neurological function. (A) Schematic diagram of IVH model establishment (injection coordinates of collagenase; ⅦS). (B, C) Comparison of hemorrhage area between different doses of collagenase (0.3U, 0.6U) (*n* = 10, **P < .01 vs 0.3U group). (D) HE staining of brain tissue at 48 h post-IVH modeling (black box indicates hemorrhage focus, scale bar = 1 cm). (E, F) Schematic diagram of lateral ventricular injection coordinates of hUC-MSCs and verification by Evans blue. (G) Longa neurological deficit score on day 3 post-modeling. (H) Righting reflex test on day 3 post-modeling. (I) Geotaxis test on day 5 post-modeling. (J) Forelimb grip test on day 5 post-modeling. **P < .01, ***P < .001 vs Sham group (*n* = 10).

### Fluorescence tracking of hUC-MSCs in IVH premature rats

Fluorescence microscopy showed that scattered fluorescent signals of hUC-MSCs were detected in the rat brain at 1 day after injection, indicating successful colonization; At day 3, both total fluorescence intensity and the distribution range of labeled cells were markedly reduced compared with day 1, without outward migration or regional expansion. By day 7, only faint punctate fluorescent residues remained near the ventricle, with overall signals nearly faded out. This progressive signal decay is mainly attributed to hUC-MSC apoptosis triggered by hemorrhagic inflammatory stress, rather than immune rejection. Neonatal rat immunity is immature at the transplantation time point, and hUC-MSCs have low immunogenicity, so obvious immune elimination did not occur.^6^ We further extended tracking to day 21 and day 32; no specific hUC-MSC fluorescent signals were captured at either late time point, indicating almost all transplanted cells lost viability and were cleared within 21 days post-injection. Collectively, these data demonstrated that hUC-MSCs can colonize and exert short-term neuroprotective effects in the IVH-injured brain for at least 7 days (Figure 3).

**Figure 3.**
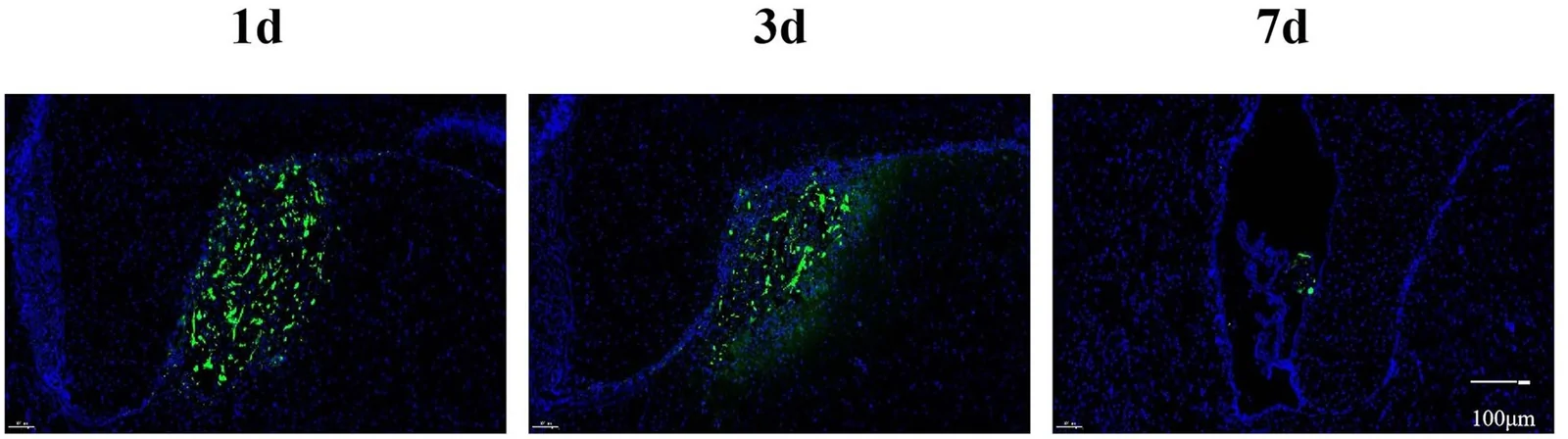
*In vivo* fluorescence tracking results of hUC-MSCs fluorescent signal distribution at 1, 3, and 7 days after injection (from left to right), showing the colonization, survival, and distribution patterns of hUC-MSCs in the IVH-injured rat brain (scale bar = 100 μm).

### Effects of hUC-MSCs on body weight and neurobehavioral function of IVH premature rats

Body weight monitoring showed no significant difference in body weight among groups on P4 (*P *> .05); on P10 and P14, the body weight of the Vehicle group (approximately 15 and 20 g, respectively) was significantly lower than that of the Sham group (approximately 20 and 25 g, respectively) (*P *< .01), while the body weight of the hUC-MSCs-treated group (approximately 18 and 23 g, respectively) was significantly higher than that of the Vehicle group (*P *< .01, Figure 4A). Neurobehavioral evaluation showed: at 7 days post-treatment, the geotaxis climbing speed of the hUC-MSCs-treated group (approximately 8 s) was significantly faster than that of the Vehicle group (approximately 15 s) (*P *< .01), and the forelimb grip time (approximately 6 s) was significantly longer than that of the Vehicle group (approximately 2 s) (*P *< .01, Figure 4B and C); at 21 days post-treatment, In the rotarod test, the fall distance of rats in the hUC-MSCs-treated group (approximately 20 cm) was significantly shorter than that of rats in the Vehicle group (approximately 40 cm); the fall velocity (approximately 100 rpm) was significantly lower than that of the Vehicle group (approximately 200 rpm); and the fall latency (approximately 250 s) was significantly longer than that of the Vehicle group (approximately 100 s) (all *P *< .01, Figure 5A-C); at 28 days post-treatment, the Morris water maze place-navigation latency (approximately 40 s) of the hUC-MSCs-treated group was significantly shorter than that of the Vehicle group (approximately 60 s) (*P *< .05), and the number of platform crossings, percentage of time spent in the target quadrant, and percentage of swimming distance in the target quadrant in the spatial probe test (approximately 3 times, 40%, and 40%, respectively) were significantly higher than those of the Vehicle group (approximately 1 time, 20%, and 20%, respectively) (all *P *< .01, Figure 5D-G), suggesting that hUC-MSCs could significantly ameliorate short- and long-term neurobehavioral deficits in premature rats with IVH, including motor coordination, muscle strength, and learning/memory abilities.

**Figure 4.**
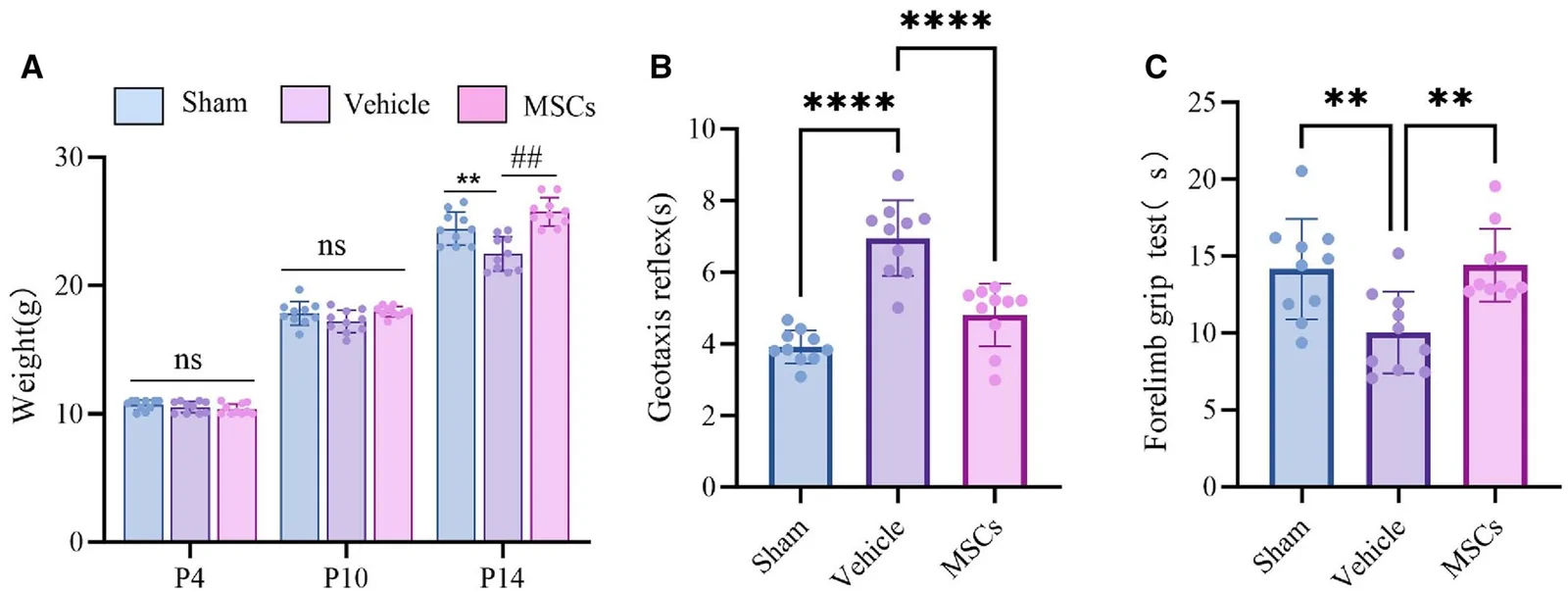
Effects of hUC-MSCs on short-term prognosis of IVH rats. (A) Body weight changes of each group on postnatal days 4, 10, and 14. (B) Geotaxis test results of each group at 7 days post-treatment. (C) Forelimb grip test results of each group at 7 days post-treatment. ns: no statistical difference; compared with Sham group, **P < .01; ****P < .000 compared with Vehicle group, ^##^*P* < .01 (*n* = 10).

**Figure 5.**
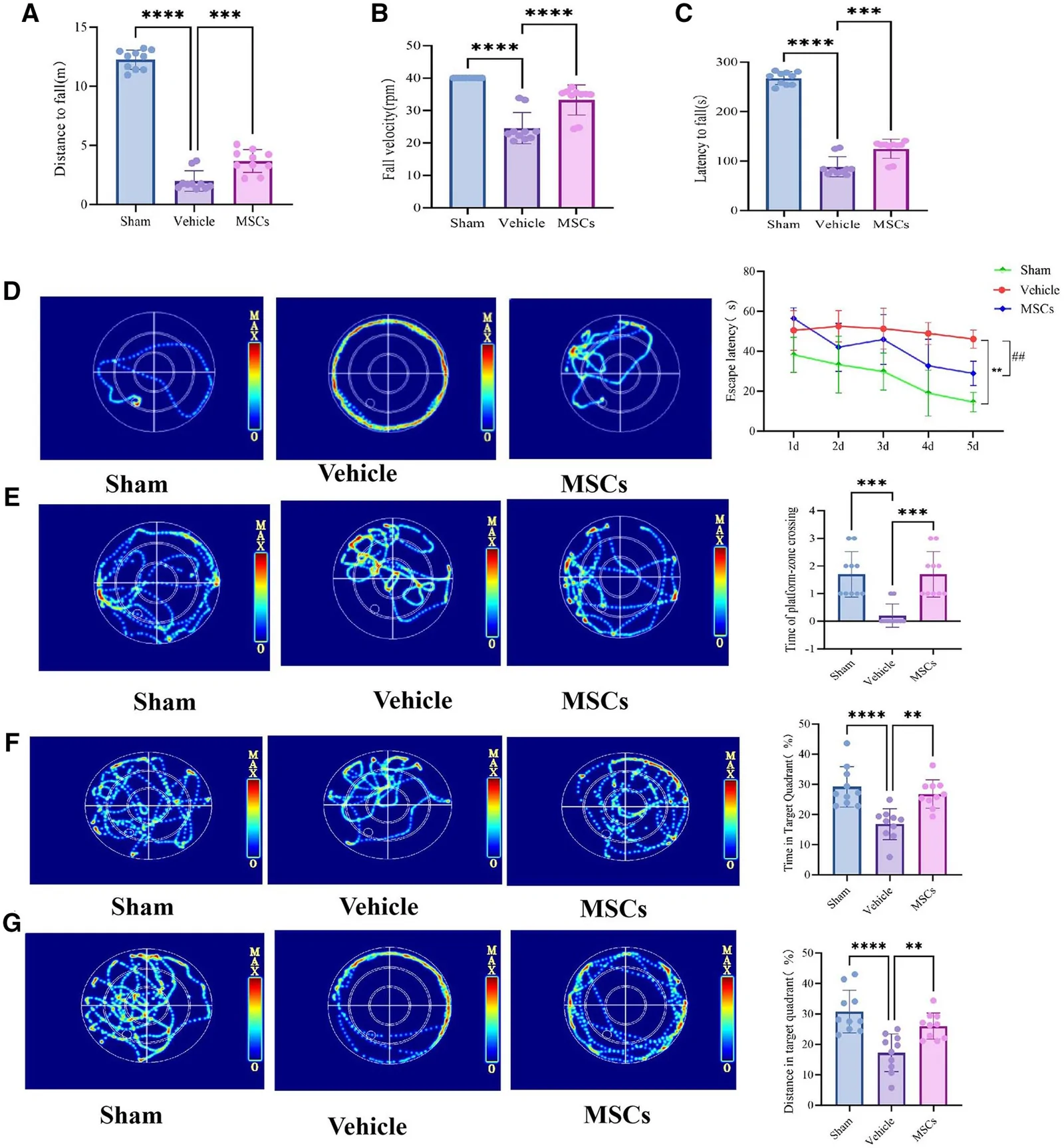
Effects of hUC-MSCs on long-term neurobehavioral function of IVH premature rats. (A-C) Treatment 21 days after, rotarod test, falling distance, falling speed, falling latency (compared with Sham, *P* < .001, ****P < .0001, *n* = 10). (D-G) Treatment 28 days, Morris water maze, time spent in target quadrant, number of platform crossings, time spent in target quadrant percentage, distance moved in target quadrant percentage (compared with Sham, **P < .01; compared with Vehicle, ^##^*P* < .01, *n* = 10).

### Effects of hUC-MSCs on brain tissue morphology and cell apoptosis of IVH premature rats

At 32 days post-treatment, HE staining showed that the right cerebral cortex of the Sham group had a complete structure and uniform thickness, with neatly arranged neurons in the classic hippocampus; the right cerebral cortex of the Vehicle group was significantly thinner, with disordered arrangement and reduced number of hippocampal neurons; compared with the Vehicle group, the hUC-MSCs-treated group exhibited significantly improved structural integrity of the cerebral cortex, as well as more ordered arrangement and a higher number of hippocampal neurons (Figure 6A and B). Nissl staining showed that the Sham group had a large number of neurons with clear Nissl bodies in the cerebral cortex and classic hippocampus; the Vehicle group had significantly reduced neurons, dissolved Nissl bodies, and obvious ventricular dilatation; the hUC-MSCs-treated group had increased neuronal survival, restored Nissl body structure, and significantly reduced ventricular dilatation (Figure 6C and D). TUNEL staining showed only a small number of apoptotic cells in the Sham group (TUNEL-positive cell rate <5%), a significant increase in apoptotic cells in the Vehicle group (TUNEL-positive cell rate >30%), and a significant decrease in apoptotic cells in the hUC-MSCs-treated group compared with the Vehicle group (TUNEL-positive cell rate <15%) (*P *< .01). Merge images of DAPI and TUNEL showed that apoptotic signals were mainly concentrated in the neuronal region, suggesting that hUC-MSCs could significantly inhibit IVH-induced neuronal apoptosis in brain tissue (Figure 6E).

**Figure 6.**
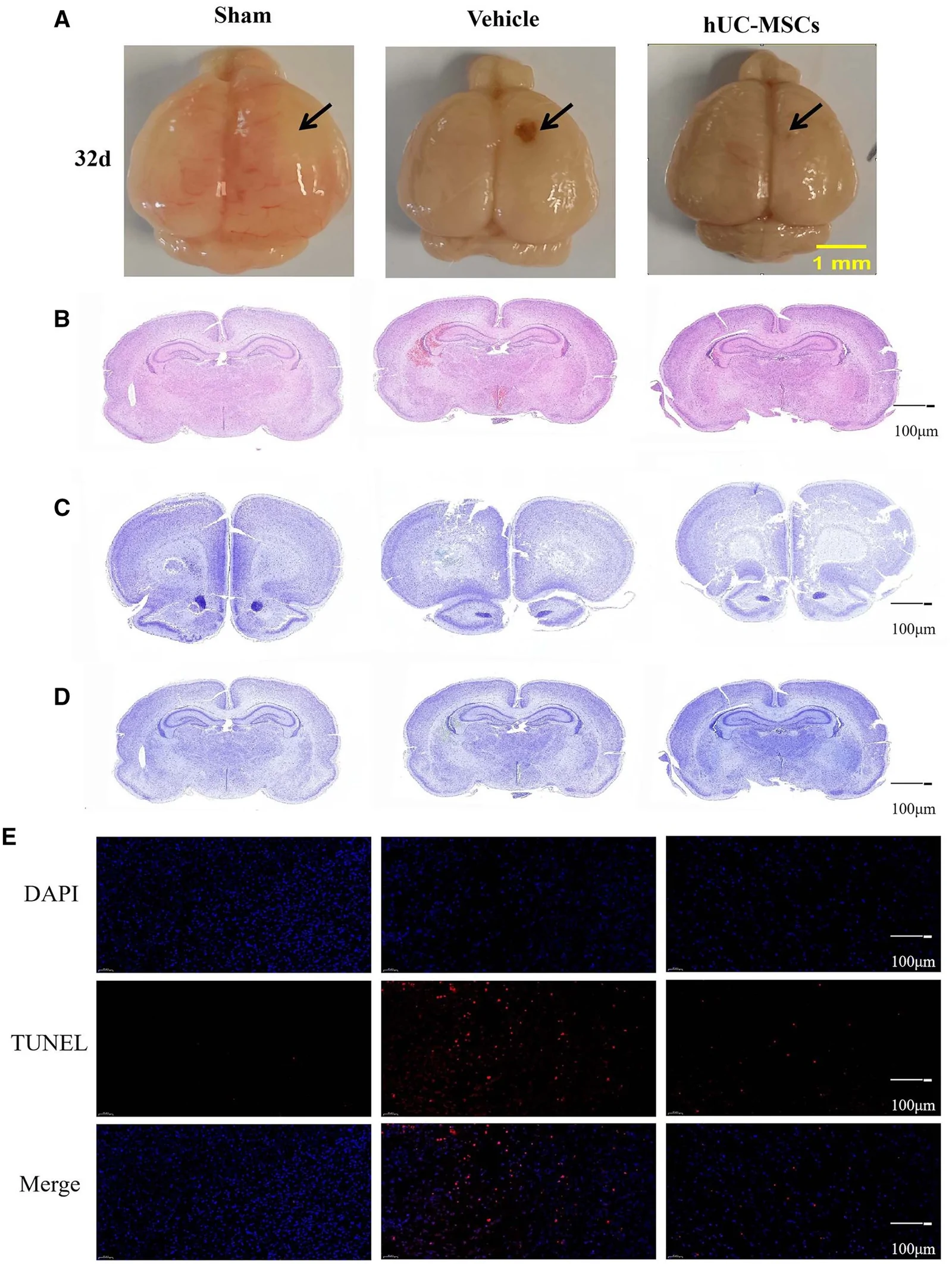
Effects of hUC-MSCs on brain tissue morphology and cell apoptosis of IVH premature rats. (A) Comparison of the right cerebral cortex of each group at 32 days post-treatment (black arrow indicates the right cerebral cortex region, scale bar = 1mm). (B) HE staining of the classic hippocampus of each group (scale bar = 100 μm). (C) Nissl staining of the cerebral cortex of each group (scale bar = 100 μm). (D) Nissl staining of the classic hippocampus of each group (scale bar = 100 μm). (E) TUNEL staining of brain tissue of each group (scale bar = 100 μm) and quantitative analysis of apoptotic cells; **P < .01, ***P < .001 vs Sham group; ^##^*P* < .01 vs Vehicle group (*n* = 3).

### Effects of hUC-MSCs on the expression of apoptosis-related proteins in IVH premature rats

WB analysis revealed that the expression of pro-apoptotic proteins Bax and cleaved caspase-3 (cleaved caspase-3/caspase-3 ratio) in the Vehicle group was significantly higher than that in the Sham group (*P *< .05 or *P *< .001), while the expression of anti-apoptotic protein Bcl2 was significantly lower than that in the Sham group (*P *< .01); after hUC-MSC administration, the relative expression of Bax protein in the hUC-MSCs-treated group was significantly downregulated compared with the Vehicle group (*P *< .01), the cleaved caspase-3/caspase-3 ratio was significantly decreased (*P *< .001), and the relative expression of Bcl2 protein was significantly upregulated (*P *< .05), suggesting that hUC-MSCs inhibit neuronal apoptosis by regulating the mitochondrial apoptotic pathway (Figure 7).

**Figure 7.**
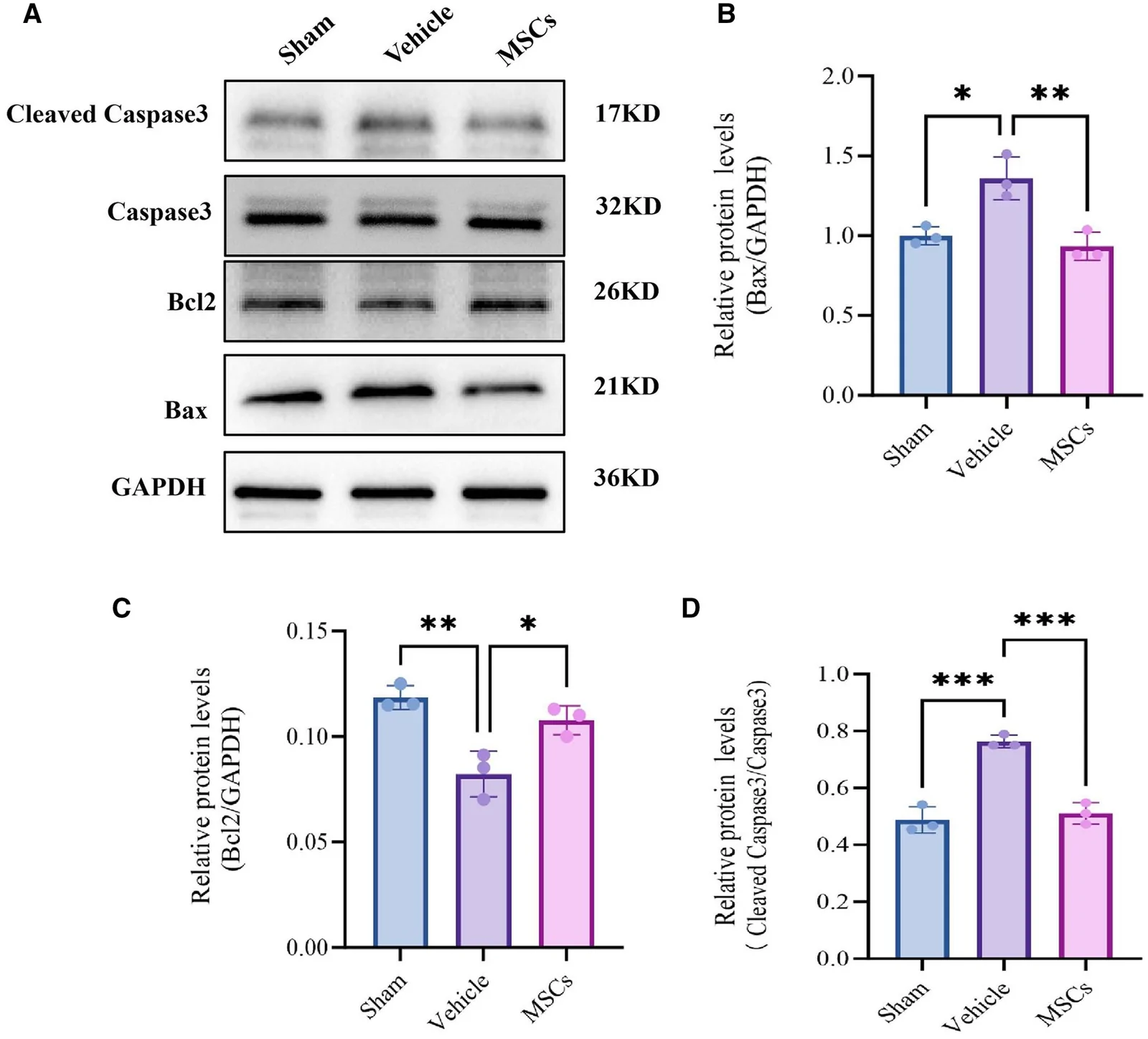
Effects of hUC-MSCs on the expression of apoptosis-related proteins in IVH premature rats. (A) Expression of Bax, Bcl2, cleaved caspase-3, and caspase-3 proteins (internal reference: GAPDH). (B-D) Quantitative analysis of Bax, Bcl2, and cleaved caspase-3/caspase-3 ratio. *P < .05, **P < .01, ***P < .001 vs Sham group; **P < .01 vs Vehicle group (*n* = 3).

### Effects of hUC-MSCs on the expression of neuroregeneration-related proteins in IVH premature rats

WB analysis revealed that the expression of astrocyte activation marker GFAP in the Vehicle group was significantly higher than that in the Sham group (*P *< .0001), while the expression of neural stem cell marker Nestin and mature neuron marker Tuj1 (relative value approximately 0.5) was significantly lower than that in the Sham group (*P *< .01 or *P *< .001); after hUC-MSC administration, the expression of GFAP in the hUC-MSCs-treated group (relative value approximately 1.0) was significantly downregulated (*P *< 0.01), and the expression of Nestin and Tuj1 was significantly upregulated (*P *< .01), suggesting that hUC-MSCs can inhibit excessive activation of astrocytes, promote the proliferation of neural stem cells and maturation of neurons, and exert neuroregenerative effects (Figure 8).

**Figure 8.**
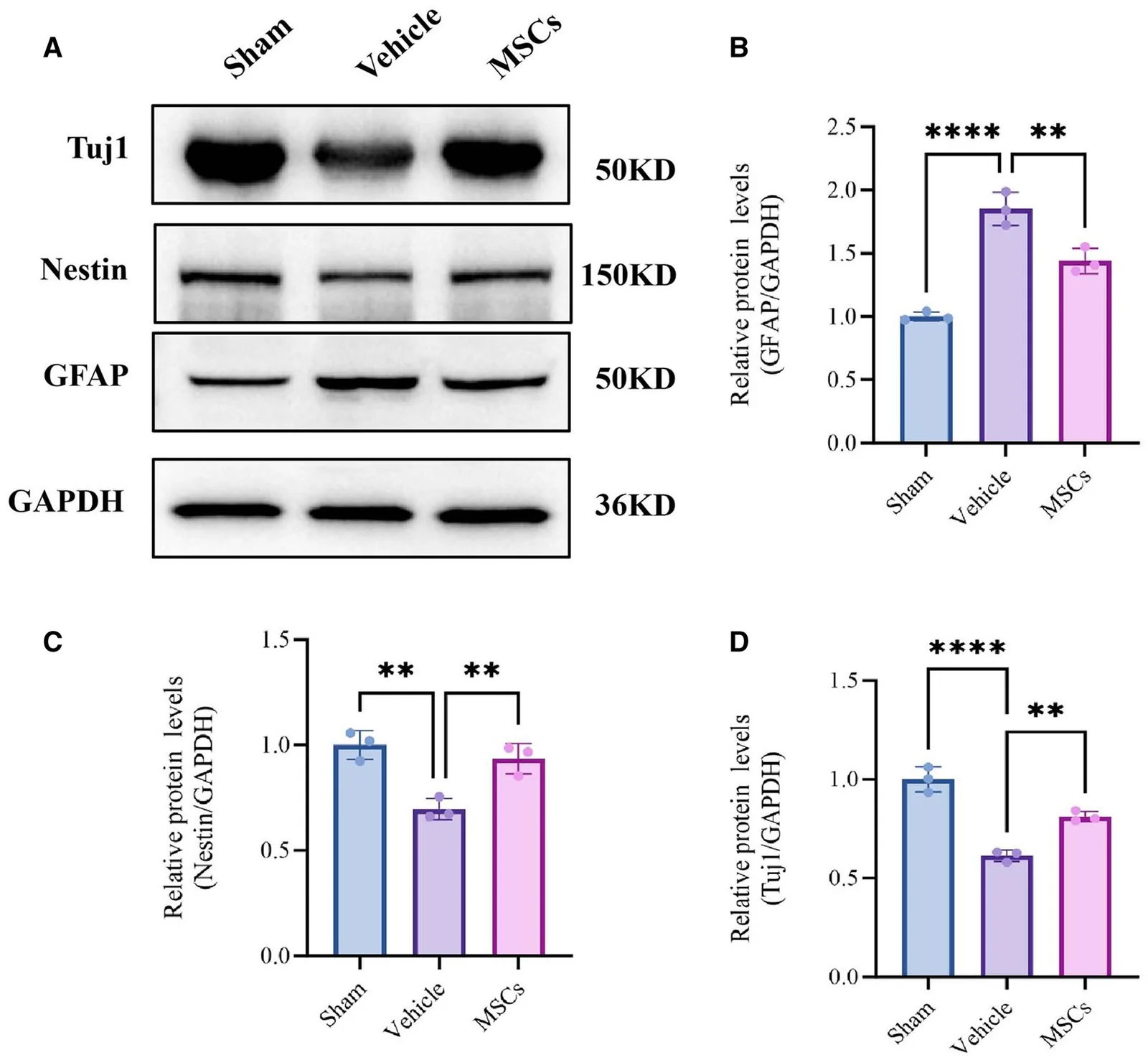
Effects of hUC-MSCs on the expression of neuroregeneration-related proteins in IVH premature rats. (A) Expression of glial fibrillary acidic protein (GFAP), Nestin, and Tuj1 proteins. (B-D) Quantitative analysis of the relative expression of glial fibrillary acidic protein (GFAP), Nestin, and Tuj1 proteins. ****P < .0001 vs Sham group; **P < .01 vs Vehicle group (*n* = 3).

### Effects of hUC-MSCs on oxidative stress in IVH premature rats

Detection of oxidative stress indicators showed that the content of lipid peroxidation marker MDA in the Vehicle group was significantly higher than that in the Sham group (*P *< .0001), while the activities of antioxidant enzymes SOD, GSH-PX, CAT, and the expression of stress-induced antioxidant enzyme HO-1 were significantly lower than those in the Sham group (*P *< .0001); after hUC-MSC administration, the MDA content in the hUC-MSCs-treated group (approximately 8 nmol/mg) was significantly decreased (*P *< .01), and the activities of SOD, GSH-PX, CAT, and the expression of HO-1 were significantly increased (*P *< .01) (Figure 9A-F), suggesting that hUC-MSCs can significantly alleviate IVH-induced oxidative stress injury by scavenging oxidative products and enhancing the function of the antioxidant system.

**Figure 9.**
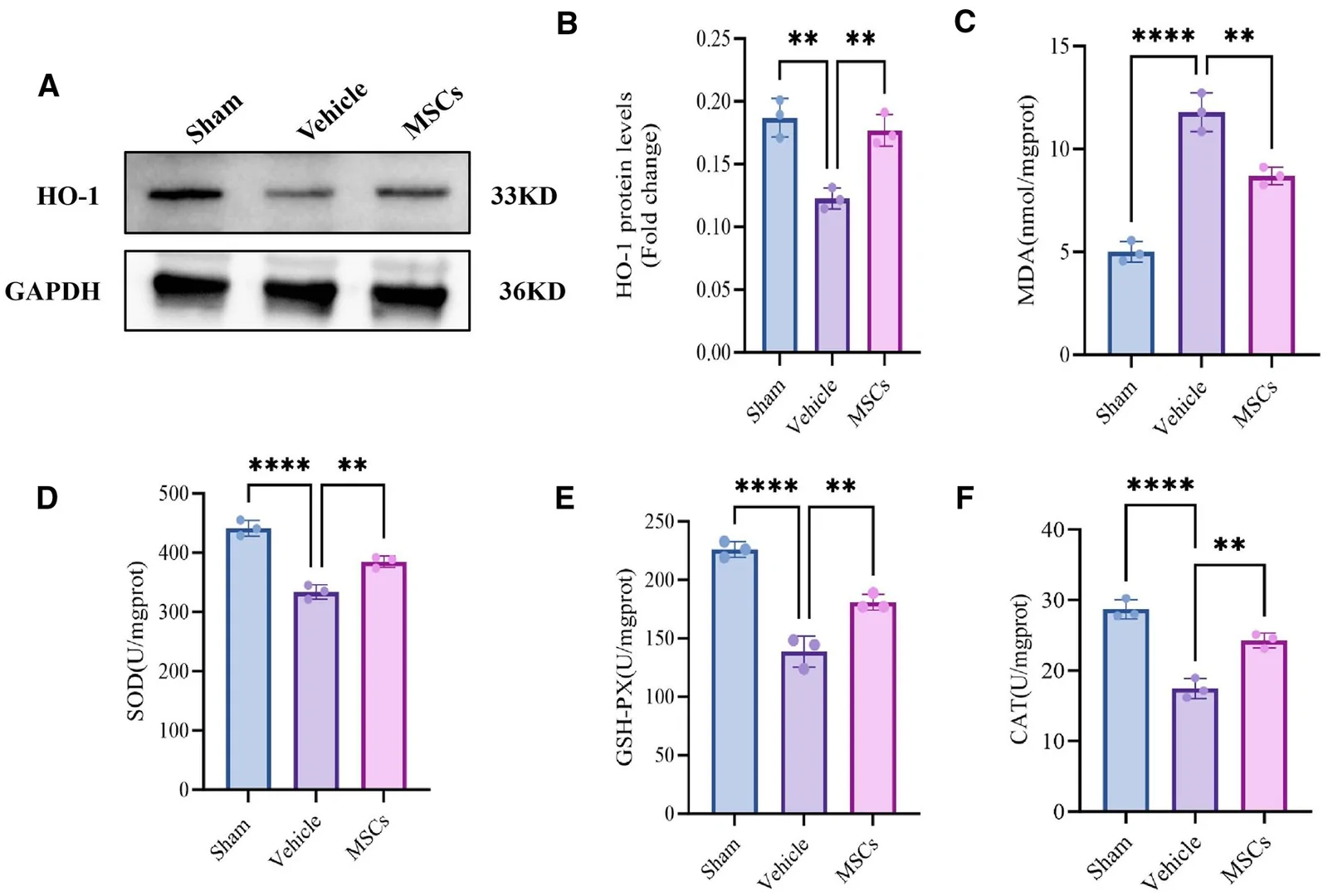
Effects of hUC-MSCs on oxidative stress in IVH premature rats. (A) WB expression of HO-1 protein. (B) Quantitative analysis of the relative expression of HO-1 protein. (C-F) Detection results of MDA content and activities of SOD, GSH-PX, and CAT. **P < .01, ****P < .0001 vs Sham group; **P < .01 vs Vehicle group (*n* = 3).

### Effects of hUC-MSCs on inflammatory response in IVH premature rats

ELISA and WB analysis revealed that the contents of pro-inflammatory factors IL-6 and TNF-α in the Vehicle group were significantly higher than those in the Sham group (Figure 10C and D), while the levels of anti-inflammatory factors IL-10 and TGF-β were significantly lower (Figure 10E and F), and the protein expression of anti-inflammatory marker Arg1 was significantly lower than that in the Sham group (*P *< .01, Figure 10A and B). After MSCs treatment, the levels of IL-6 and TNF-α were significantly downregulated (*P *< .01 or *P *< .0001), and the levels of IL-10, TGF-β as well as the expression of Arg1 were significantly upregulated (*P *< .01 or *P *< .0001). These results suggest that hUC-MSCs can inhibit the inflammatory cascade reaction after IVH by downregulating pro-inflammatory factors and upregulating anti-inflammatory markers (Figure 10A-F).

**Figure 10.**
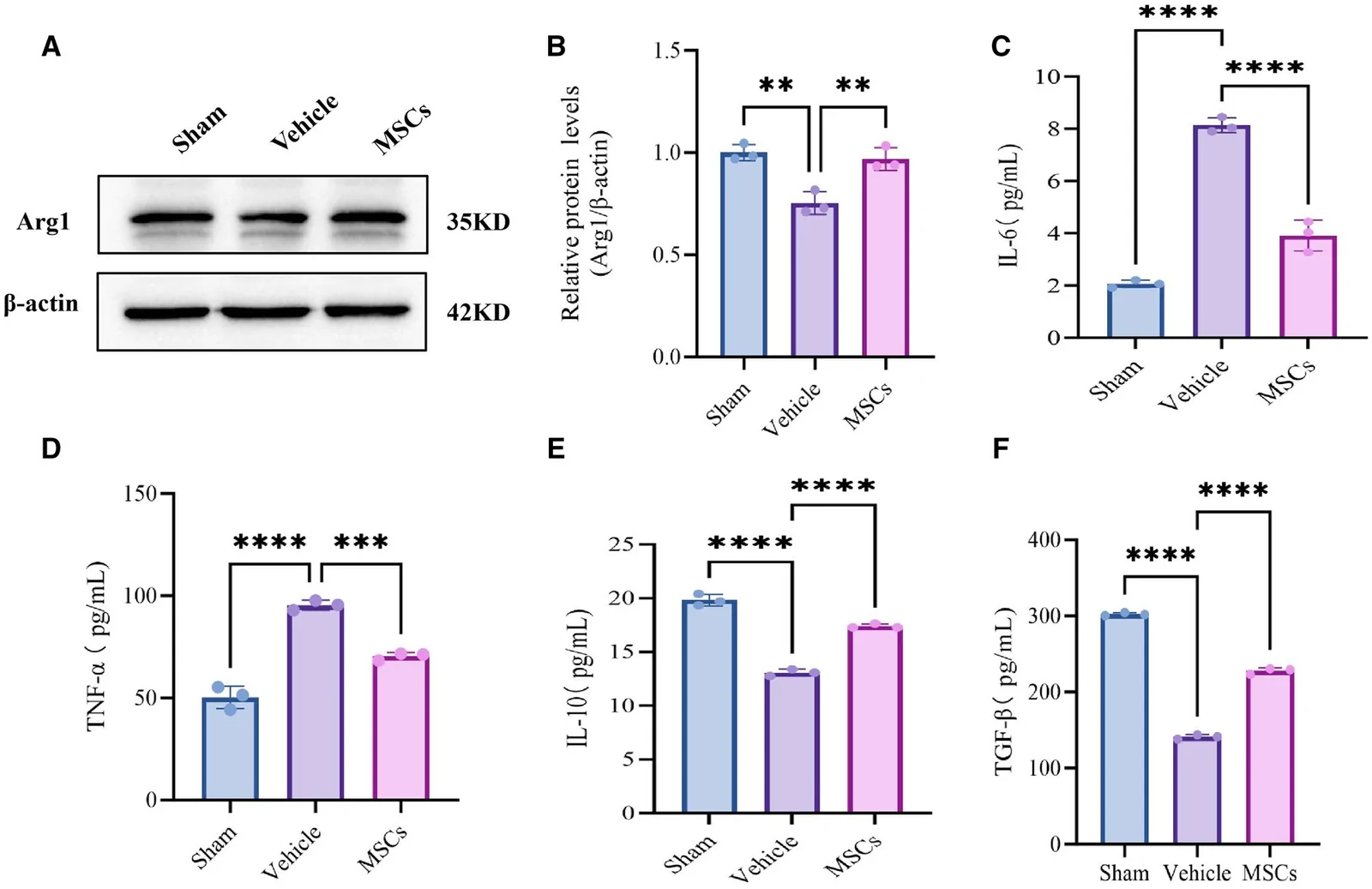
Effects of hUC-MSCs on inflammatory response in preterm rats with IVHA. (A) Arg1 protein expression. (B) Quantitative analysis of relative Arg1 protein expression. (C, D) Detection results of IL-6 and TNF-α contents. (E, F) Detection results of IL-10 and TGF-β contents. **P < .01; ***P < .001, ****P < .0001, vs Sham group; ****P < .0001 vs Vehicle group (*n* = 3).

### Bioinformatics analysis results

A total of 1134 common targets were identified by screening the intersecting targets between hUC-MSCs and IVH, 83 unique targets for hUC-MSCs and 28 unique targets for IVH (Figure 11A). PPI network analysis showed that BDNF, TrkB, CREB, AKT, IL-6, TNF-α, HO-1, Arg1, NLRP3, and HMOX1 were core targets, with tight interactions among them (Figure 11B). GO functional enrichment analysis revealed that BP were mainly involved in transcriptional regulation (positive regulation of DNA-templated transcription, negative regulation of gene expression), apoptosis inhibition, neuroregeneration, etc. CC were primarily enriched in the nucleus, extracellular region, protein complex, etc. MF were dominated by transcription factor binding, growth factor activity, cytokine activity, etc. (Figure 11C). KEGG pathway analysis indicated that the differential targets were mainly enriched in the PI3K-Akt, HIF-1, MAPK, and other signaling pathways (Figure 11D), which provided a core direction for subsequent verification of molecular mechanisms.

**Figure 11.**
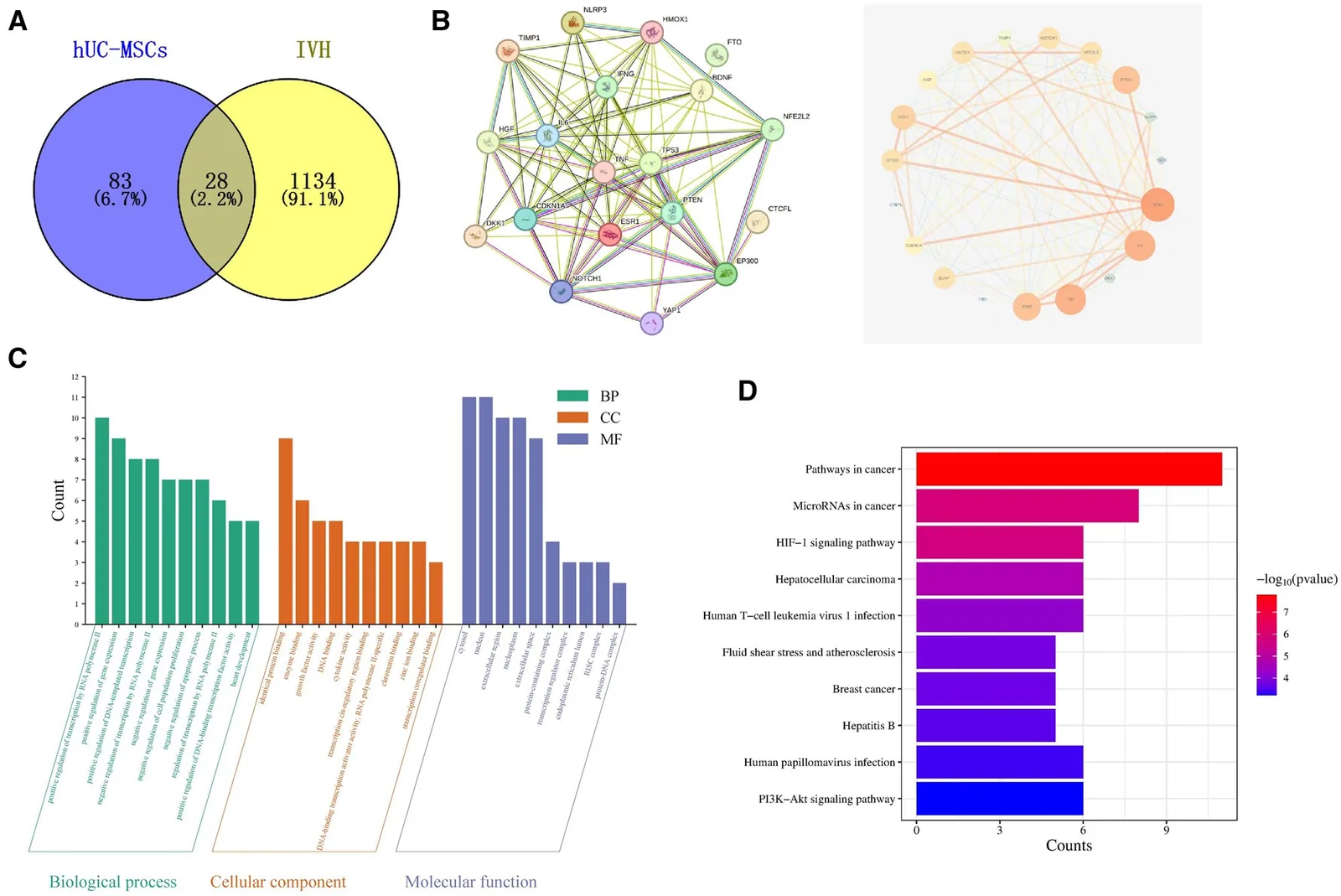
Bioinformatics analysis of hUC-MSCs and IVH. (A) The Venn diagram of the intersection targets hUC-MSCs and IVH, a total of 1134 common targets were screened out, 83 specific targets of hUC-MSCs, and 28 specific targets of IVH. (B) The PPI network of core targets there is a close interaction between core targets. (C) GO function enrichment analysis of intersection targets, biological processes (BP) mainly involve transcriptional regulation, apoptosis inhibition, regeneration, etc., cellular components (CC) are enriched in the nucleus, protein complexes, etc., and molecular functions (MF) are mainly transcription factor binding, growth factor, etc. (D) KEGG signal pathway enrichment analysis of intersection targets, different targets are mainly enriched in PI3K-Akt, HIF-1, and other pathways.

### Effects of hUC-MSCs on neurotrophic factors and signaling pathways in IVH premature rats

#### BDNF/TrkB pathway

RT-PCR and WB analysis revealed that the BDNF gene transcription and protein expression, TrkB gene transcription level, and p-TrkB/TrkB ratio in the Vehicle group were significantly lower than those in the Sham group (*P *< .001 or *P *< .01). After MSCs treatment, the relative expression level of *BDNF* mRNA was significantly upregulated compared with the Vehicle group (*P *< .01, Figure 12C), the relative expression level of BDNF protein was significantly increased compared with the Vehicle group (*P *< .01, Figure 12A and B), the relative expression level of *TrkB* mRNA was significantly elevated compared with the Vehicle group (*P *< .01, Figure 12F), and the p-TrkB/TrkB ratio was significantly higher than that in the Vehicle group (*P *< .01, Figure 12D and E). These results suggest that hUC-MSCs can activate the brain-derived neurotrophic factor and its receptor pathway.

**12.**
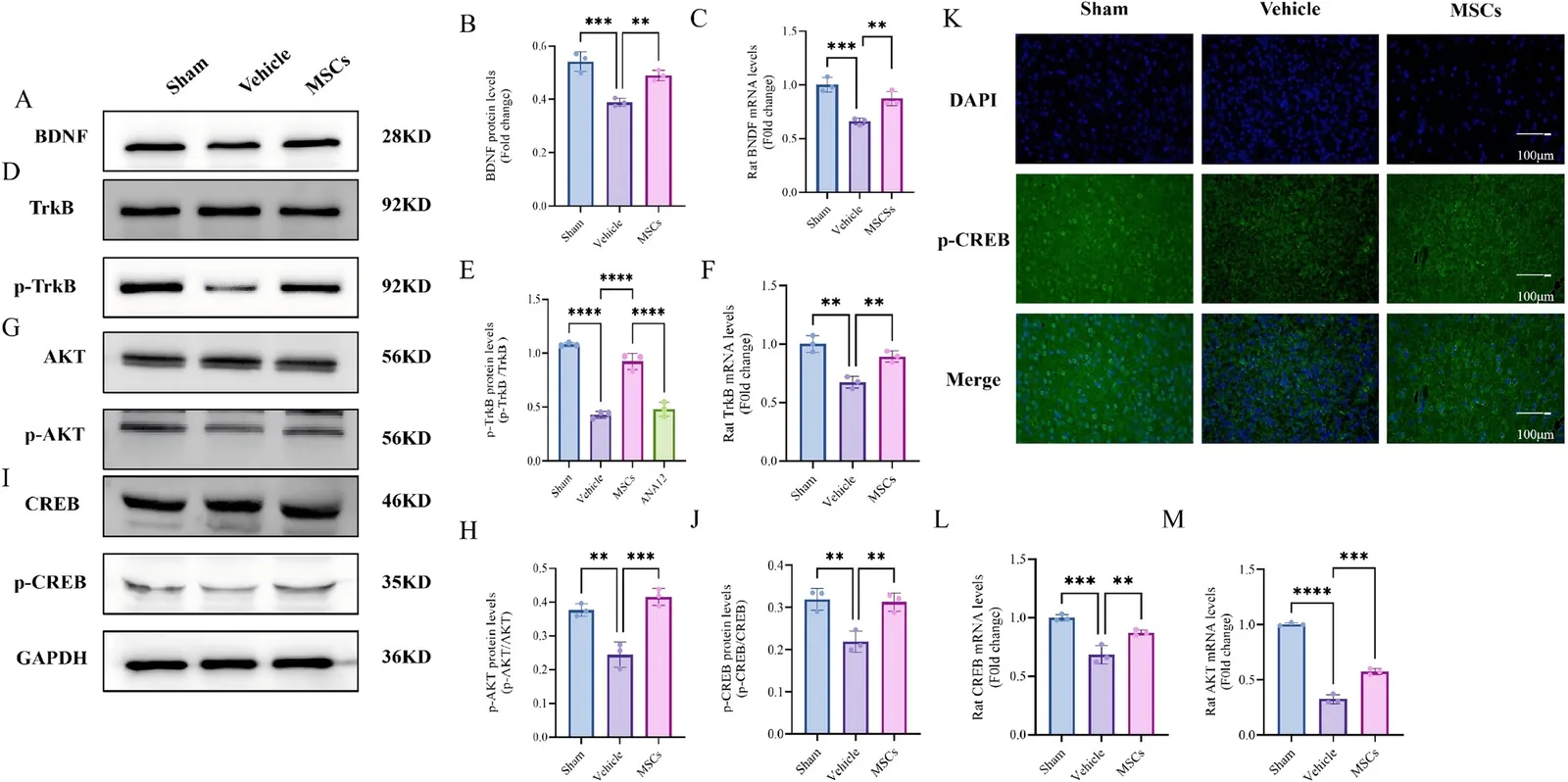
Activation of BDNF/TrkB/CREB/PI3K-Akt signaling pathway by hUC-Cs. (A, D, G, I) Western blot bands of BDNF, TrkB, AKT, CREB, and phosphoproteins. (B, E, H, J) Quantitative analysis of the relative expression levels of BDNF, p-TrkB/TrkB, p-AKT/AKT, and pCREB/CREB proteins (***P < .001, ****P < .0001 vs. Sham group; **P < .01, *n* = 3 vs. Vehicle group). (C, F, L, M) Quantitative analysis of the relative expression levels of BDNF, TrkB, CREB, AKT mRNA (***P < .001 vs. Sham group; **P < .01, *n* = 3. Vehicle group). (K) Immunofluorescence staining of p-CREB protein (scale bar = 100 μm), indicating enhanced intracellular localization expression of p-CREB in neurons.

#### CREB/PI3K-Akt pathway

WB and immunofluorescence results showed that the ratios of p-CREB/CREB, p-AKT/AKT, and *AKT* mRNA in the Vehicle group were significantly lower than those in the Sham group (*P *< .01, Figure 12G-J), and immunofluorescence revealed weak fluorescent signals of p-CREB in the Vehicle group (Figure 12K). After MSCs treatment, the p-CREB/CREB and AKT mRNA ratio was significantly increased compared with the Vehicle group (*P *< .01), and the p-AKT/AKT ratio was also significantly elevated compared with the Vehicle group (*P *< .01). RT-PCR results showed that the transcriptional level of the CREB gene was synchronously upregulated compared with the Vehicle group (*P *< .01, Figure 12L). Immunofluorescence demonstrated that the fluorescent signals of p-CREB in the hUC-MSCs-treated group were enhanced and mainly localized in the nuclei of neurons. These results suggest that hUC-MSCs can activate the CREB and PI3K-Akt signaling pathways.

#### Signaling pathway blocking experiment

After TrkB blockade with ANA12, the regulatory effects of hUC-MSCs on apoptosis, neuroregeneration, and signaling pathways were significantly reversed: compared with the hUC-MSCs-treated group alone, the ANA12 group showed upregulated Bax expression, downregulated Bcl2 expression, downregulated Nestin and Tuj1 expression, upregulated GFAP expression, and significantly decreased expression of BDNF, p-CREB, p-TrkB, and p-AKT (*P *< .01), confirming that BDNF/TrkB is the core pathway for hUC-MSCs to exert protective effects, and it regulates the CREB and PI3K-Akt pathways downstream (Figure 13).

**Figure 13.**
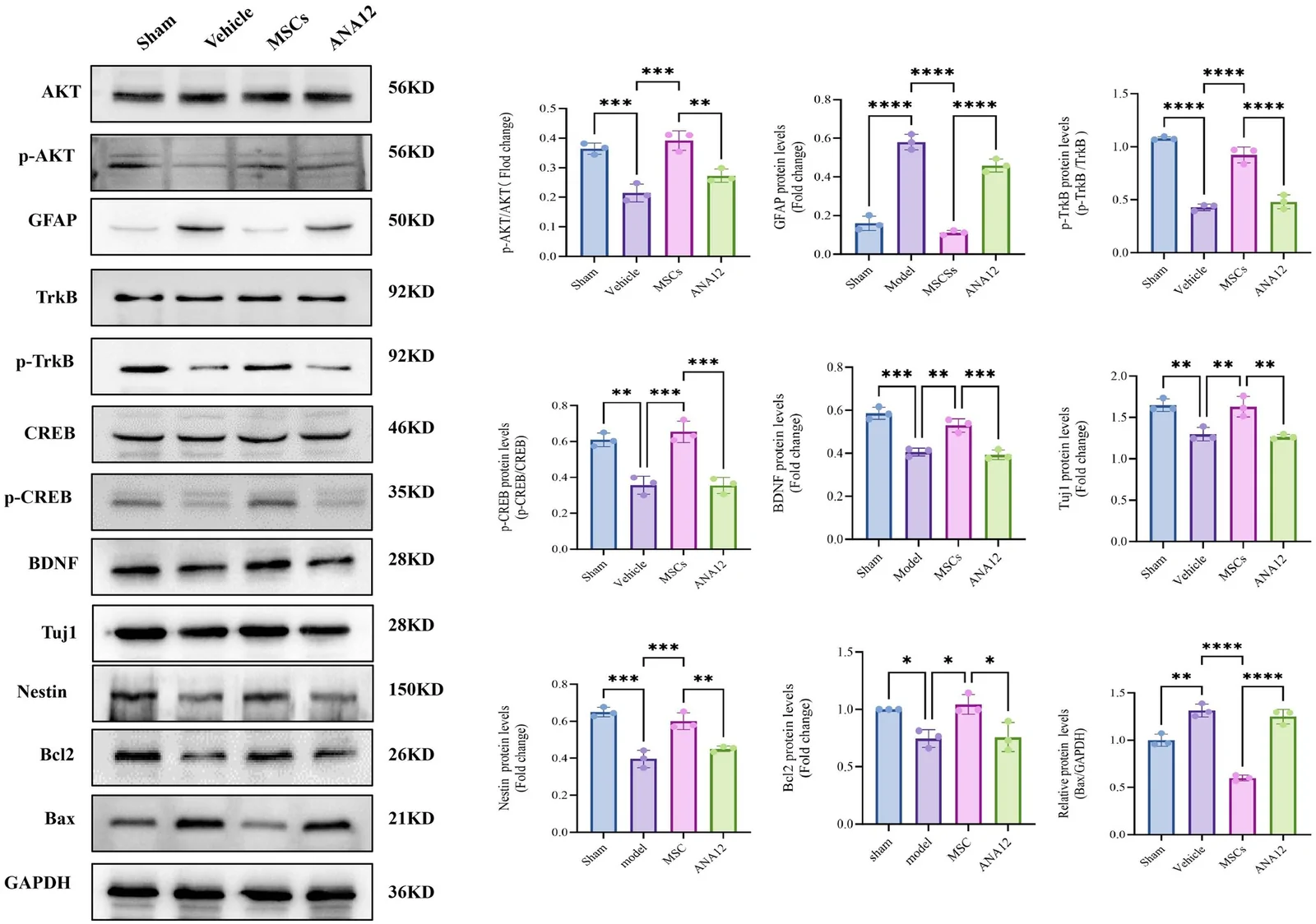
Reversal effect of TrkB antagonist ANA12 on the neuroprotective effect of hUC-MSCs. After TrkB by ANA12, the BDNF/TrkB/CREB/PI3K-Akt pathway and the expression of downstream apoptosis-neuroregeneration-related proteins (AKT, p-AKT, TrkB, p-TrkB, CREB, p-CREB, BDNF Tuj1, Nestin, Bcl2, Bax, GFAP) were significantly reversed (*P < .05, **P < .01, ***P < .001, ****P < .0001, *n* = 3).

## Discussion

As a life-threatening complication of central nervous system injury in premature infants, IVH has a pathophysiological process centered on the “ischemia-hypoxia initiation-inflammatory cascade amplification-oxidative stress imbalance-uncontrolled neuronal apoptosis.” All links are interconnected and synergistically aggravate brain injury, ultimately leading to irreversible neurological sequelae.^9^^,^^10^ Currently, there is no effective clinical intervention, and cell transplantation has become a potential strategy to overcome this dilemma. Among them, hUC-MSCs have become a research hotspot in the field of neuroprotective repair due to their convenient source, low immunogenicity, and powerful paracrine functions. Through systematic *in vivo* experiments and bioinformatics analysis, this study clarified the neuroprotective effects of hUC-MSCs on IVH premature rats and deeply elucidated the core mechanism of their multi-dimensional and multi-pathway synergistic regulation, thus filling the gaps in current research and providing more robust experimental evidence for clinical translation.

This study was the first to confirm through fluorescence tracking experiments that hUC-MSCs can colonize the brain of IVH premature rats at 1 day after lateral ventricular injection, contrary to our initial description, fluorescence intensity and cell distribution range declined markedly at day 3, with only residual weak signals observable at day 7, and no positive signals could be identified at day 21 and day 32. A potential cross-species compatibility concern was addressed here: hUC-MSCs exert neuroprotection mainly via evolutionarily conserved paracrine factors (BDNF, TGF-β, antioxidant enzymes) rather than species-specific cell-surface receptor binding. These bioactive molecules share high homology between humans and rats to activate TrkB/PI3K-Akt cascades. Experiments using 3 independent umbilical cord donors produced identical therapeutic outcomes, ruling out random donor-specific effects. Notably, P4 neonatal rats possess immature immune systems with minimal mature T/B cell activity, so robust xenogeneic rejection does not occur within our 7-day observation window.^8^ Meanwhile, hUC-MSCs themselves have low immunogenicity and strong immunosuppressive paracrine capacity to further suppress local inflammatory reactions. Control groups injected with equal volume human somatic cells showed no neuroprotective phenotype, verifying the observed benefits are hUC-MSC-specific instead of a universal xenogeneic immune reaction. Clinically, this rodent xenograft model serves only for preliminary mechanism exploration of MSC paracrine pathways; future clinical translation will rely on autologous/allogeneic human hUC-MSC infusion without cross-species barriers. The gradual reduction of labeled cells is driven by apoptosis induced by the hostile post-IVH microenvironment instead of immune rejection.^14^ Although grafted cells undergo progressive loss within 1 week, their paracrine factors secreted during the 7-day survival window continuously remodel the intracerebral microenvironment, which lays the foundation for long-term neurological recovery.^11^^,^^12^ Combined with the subsequent experimental results, the survival of hUC-MSCs in the brain for 7 days is not solely dependent on their intrinsic anti-apoptotic capacity, but is closely associated with the amelioration of the injured brain microenvironment via their paracrine activity. The results of this study showed that after hUC-MSC administration, the levels of pro-inflammatory factors IL-6 and TNF-α in the brain were significantly downregulated, the expression of anti-inflammatory marker Arg1 was upregulated, the content of lipid peroxidation marker MDA was decreased, and the activities of antioxidant enzymes SOD, GSH-PX, and CAT were increased. The formation of this anti-inflammatory and anti-oxidative microenvironment effectively reduces the toxic effects of damaging factors on hUC-MSCs, providing favorable conditions for their colonization and survival.^12^^,^^13^ This is consistent with the conclusion in existing studies that “the survival of MSCs depends on the host tissue microenvironment.”^14^^,^^15^ However, this study further clarified the specific survival duration of hUC-MSCs in the brain of IVH premature rats (at least 7 days) and initially revealed the mutual regulatory relationship between microenvironment improvement and cell survival, filling the gap in the unclear colonization pattern of hUC-MSCs in the IVH brain in existing studies.

Cell apoptosis is a core pathological link of secondary brain injury after IVH. This study directly confirmed through TUNEL staining that the number of apoptotic cells in the brain tissue of the IVH model group (Vehicle group) increased significantly, and apoptotic signals were mainly concentrated in the neuronal region (shown by DAPI/TUNEL Merge images), which is consistent with the pathological characteristic that neurons are vulnerable to ischemia-hypoxia, inflammation, and oxidative stress injury after IVH.^16^ After hUC-MSC administration, the number of apoptotic cells decreased significantly, and WB analysis revealed that the pro-apoptotic proteins Bax and cleaved caspase-3 (cleaved caspase-3/caspase-3 ratio) were significantly downregulated, while the anti-apoptotic protein Bcl-2 was significantly upregulated, clarifying the core mechanism by which hUC-MSCs inhibit neuronal apoptosis by regulating the mitochondrial apoptotic pathway.^17^^,^^18^ In-depth analysis shows that the regulation of apoptosis by hUC-MSCs is not a single target effect but synergistic with their anti-inflammatory and anti-oxidative effects: on the one hand, inflammatory factors IL-6 and TNF-α can activate the NF-κB pathway to promote Bax expression and inhibit Bcl-2 expression, and hUC-MSCs can indirectly block this pro-apoptotic pathway by downregulating the levels of IL-6 and TNF-α.^13^^,^^19^ On the other hand, ROS produced by oxidative stress can directly damage mitochondrial membrane potential and promote caspase-3 activation, while hUC-MSCs activate the antioxidant system composed of SOD, GSH-PX, CAT, and HO-1 to scavenge ROS and reduce mitochondrial damage, thereby directly inhibiting the caspase-3-mediated apoptotic cascade.^20^ This “dual anti-apoptotic effect of directly regulating the apoptotic pathway and indirectly blocking upstream injury triggers” is more advantageous than single anti-apoptotic drugs (such as Bcl-2 agonists), and also explains the experimental results that hUC-MSCs can significantly reduce neuronal apoptosis and improve brain tissue structure injury in this study.^21^^,^^22^

Neuroregeneration disorders and brain microenvironment disorders are key reasons for the difficulty in neurological function recovery after IVH. This study confirmed the promoting effect of hUC-MSCs on neuroregeneration and the improvement effect on the brain microenvironment from both histological and molecular levels through HE staining, Nissl staining, and WB detection.^23^ HE staining results showed that hUC-MSC administration could significantly improve the morphological damage such as right cerebral cortex thinning and hippocampal neuron arrangement disorder caused by IVH; Nissl staining showed that it could increase the number of neurons in the cerebral cortex and hippocampus, restore Nissl body structure, and reduce ventricular dilatation; WB analysis revealed that it could upregulate the expression of neural stem cell marker Nestin and mature neuron marker Tuj1, and downregulate the expression of astrocyte activation marker GFAP. In-depth analysis of its mechanism shows that the significant downregulation of GFAP protein expression is of great significance. after IVH, excessive activation of astrocytes will secrete a large amount of chondroitin sulfate proteoglycan, GFAP, and other substances to form glial scars, which not only physically block neural axon regeneration but also inhibit the proliferation and differentiation of neural stem cells. By downregulating GFAP expression, hUC-MSCs can not only directly inhibit glial scar formation but also reduce the neuroregeneration inhibitory factors secreted by glial scars, creating favorable conditions for neural stem cell proliferation, neuron maturation, and neural axon regeneration. At the same time, the upregulation of Nestin by hUC-MSCs suggests that it can promote the proliferation of endogenous neural stem cells, and the upregulation of Tuj1 suggests that the proliferated neural stem cells can differentiate into mature neurons smoothly. Combined with its anti-apoptotic effect, it forms a complete neuroregeneration regulatory chain of “promoting neural stem cell proliferation-inducing neuron maturation-protecting the survival of newborn neurons,” which is also the core cellular basis for hUC-MSCs to significantly improve the short-term (geotaxis, forelimb grip) and long-term (rotarod, Morris water maze) neurobehavioral functions of IVH premature rats. In addition, the result that hUC-MSCs upregulate the anti-inflammatory marker Arg1 further confirms that it can reshape the anti-inflammatory microenvironment in the brain, reduce the inhibitory effect of inflammation on neuroregeneration,^24^ and form a synergy with GFAP downregulation and neuroregeneration marker upregulation to jointly promote the transformation of the brain microenvironment in a direction conducive to neural repair.

To elucidate the core molecular mechanism underlying the neuroprotective effects of hUC-MSCs, the present study first identified 1134 intersecting targets between hUC-MSCs and IVH via bioinformatics analysis. The core targets included BDNF, TrkB, CREB, AKT, IL-6, TNF-α, HO-1, and Arg1. KEGG pathway enrichment revealed significant enrichment in the PI3K-Akt, HIF-1, and MAPK signaling pathways, while GO functional enrichment was mainly involved in biological processes such as transcriptional regulation, apoptosis inhibition, and neuroregeneration. These results provided a clear direction for subsequent verification of molecular mechanisms. On this basis, the core targets and pathways predicted by bioinformatics were comprehensively verified using molecular biological assays and signaling pathway blockade experiments. The present study confirmed that the BDNF/TrkB/CREB/PI3K-Akt pathway serves as the core signaling pathway mediating the neuroprotective effects of hUC-MSCs, and further revealed the crosstalk regulatory mechanism among these pathways.^25^ RT-PCR and WB analysis revealed that hUC-MSC administration could significantly upregulate the transcriptional and protein expression of BDNF, transcriptional level of TrkB, and activate TrkB (increased p-TrkB/TrkB ratio), CREB (increased p-CREB/CREB ratio), and PI3K-Akt pathways (increased p-AKT/AKT ratio); immunofluorescence further confirmed that p-CREB was mainly localized in the nuclei of neurons, consistent with the function of CREB as a transcription factor; after blockade with the TrkB-specific antagonist ANA12, the regulatory effects of hUC-MSCs on apoptosis (Bax downregulation, Bcl-2 upregulation), neuroregeneration (Nestin, Tuj1 upregulation), signaling pathways (p-CREB, p-AKT increase), and glial activation (GFAP downregulation) were significantly reversed, confirming that TrkB is a key mediator for hUC-MSCs to exert protective effects.^26^ In-depth analysis of the synergistic relationship between pathways shows that after BDNF (a neurotrophic factor) binds to TrkB, it can exert effects through 2 downstream pathways: one is to activate CREB to promote the transcription of neurotrophic factors and anti-apoptotic genes such as BDNF and Bcl-2, forming a “BDNF-TrkB-CREB” positive feedback regulatory loop to amplify the neuroprotective effect;^27^^,^^28^ the other is to activate the PI3K-Akt pathway to further strengthen the anti-apoptotic effect (inhibiting Bax expression, promoting Bcl-2 expression) and neuroregenerative effect (promoting neural stem cell proliferation and differentiation), forming cross-regulation with the CREB pathway. This cross-regulatory mechanism enables hUC-MSCs to achieve multi-dimensional synergistic regulation of apoptosis, inflammation, oxidative stress, and neuroregeneration through a single core pathway, explaining the molecular basis of their pleiotropic protective effects.^29^

## Conclusion

Based on systematic experimental data, this study deeply elucidated the neuroprotective effects and molecular mechanisms of hUC-MSCs on IVH premature rats. After lateral ventricular injection, hUC-MSCs can colonize and survive in the brain for at least 7 days. By activating the core BDNF/TrkB/CREB/PI3K-Akt signaling pathway, hUC-MSCs synergistically modulate multiple pathological processes, including anti-apoptosis (upregulation of Bcl-2 and downregulation of Bax and cleaved caspase-3), anti-inflammation (downregulation of IL-6 and TNF-α and upregulation of Arg1), anti-oxidation (downregulating MDA, upregulating SOD, GSH-PX, CAT, and HO-1), and neuroregeneration (upregulating Nestin and Tuj1, downregulating GFAP), thereby improving the injured brain microenvironment, repairing damaged brain tissue structure, and promoting neurological function recovery. This study provides key experimental evidence (including immunofluorescence localization and TrkB pathway blockade validation) for the colonization pattern of hUC-MSCs in the IVH-injured brain, specific neuronal protection, and multi-pathway synergy, highlights their clinical transformation advantages, and clarifies future research directions, providing more comprehensive and in-depth theoretical support and experimental basis for the clinical application of hUC-MSCs in the treatment of IVH in premature infants, with significant translational medical value.

## Data Availability

The data for this article can be accessed from the corresponding author.
